# Heart rate variability reflects endoscopic activity and systemic inflammation in patients with Crohn’s disease

**DOI:** 10.3389/fmed.2025.1746064

**Published:** 2026-01-13

**Authors:** Ruohan Wang, Fengfeng Yan, Hongjie Zhang

**Affiliations:** 1 Department of Infectious Disease and Liver Disease, The Second Hospital of Nanjing, Nanjing, China; 2 Department of Gastroenterology, Nantong Third People's Hospital, Nantong University, Nantong, China; 3 Department of Gastroenterology, First Affiliated Hospital of Nanjing Medical University, Nanjing, China

**Keywords:** Crohn’s disease, disease activity, endoscopic activity, heart rate variability, parasympathetic activity

## Abstract

**Purpose:**

It is well known that the autonomic nervous system (ANS) activity is impaired in patients with Crohn’s disease (CD). Heart rate variability (HRV), a non-invasive and repeatable measurement, was used to assess ANS activity. The relationships between disease activity and HRV need to be elucidated. Our study also aimed to explore the relationship between HRV parameters and systemic inflammatory biomarkers, and to establish an assessment model using HRV, a non-invasive tool, to assess endoscopic activity in CD.

**Patients and methods:**

A total of 84 CD patients (44 active CD and 40 in remission) were enrolled at The First Affiliated Hospital of Nanjing Medical University from January 2022 to December 2022. All patients underwent HRV examination and endoscopy. Clinical data, including demographic variables, disease activity and course, medications, and laboratory data, were collected. Binary logistic regression analysis was used to identify the factors associated with endoscopic activity, based on which the nomogram model was established.

**Results:**

Compared with patients in endoscopic remission, active CD patients had significantly ratio of low frequency to high frequency (LF/HF) (*p* < 0.001), lower standard deviation of all normal–normal (SDNN) intervals (*p* = 0.002), and high-frequency HRV (HF) (*p* = 0.011), suggesting that parasympathetic activity was reduced in active CD patients. In addition, SDNN and HF were negatively correlated with the simple endoscopic score for CD (SES-CD). Meanwhile, HR and LF/HF were found to be independent risk factors for endoscopic disease activity by univariate and binary logistic regression. A regression model was established: HRV assessment of endoscopic active = −8.335 + 0.918LF/HF + 0.084HR (C-index: 0.803, 95% confidence interval [CI]:0.710–0.896).

**Conclusion:**

The parasympathetic activity was reduced in active CD patients. Furthermore, the significant correlations between HRV parameters and inflammatory biomarkers underscore that HRV not only reflects endoscopic activity but also mirrors the systemic inflammatory burden in CD. Thus, HRV can potentially be developed into a simple, non-invasive tool for comprehensively assessing disease activity and inflammation in CD.

## Introduction

Crohn’s disease (CD) is a chronic inflammatory disorder with recurrent attacks and remission caused by the abnormality of the gastrointestinal mucosal immune system (1). Due to the repeated recurrence of the disease, blood tests, imaging, and endoscopic examination are often required, which not only impose an economic burden on patients but also seriously affect their quality of life. Therefore, it is particularly important to seek a simple and non-invasive way of disease monitoring to reduce the disease burden for patients.

There were higher levels of sympathetic activity and lower levels of parasympathetic activity in patients with CD, compared with healthy controls (2). Recent studies have shown that there is bidirectional regulation between the autonomic nervous system (ANS) and the immune system (3), with the parasympathetic nerve playing an important role (4).

Heart rate variability (HRV) reflects the continuous oscillations of the R-R intervals between normal heartbeats, regulated by the combined effects of sympathetic and parasympathetic nerves (5, 6). It has been confirmed that the sympathetic activity was dominant, while the parasympathetic activity was inhibited in CD (7). It was reported that patients with inflammatory bowel disease in clinical remission have lower parasympathetic activity than healthy controls, reflected by decreased high-frequency (HF) (8). Meanwhile, Coruzzi et al. revealed the impairment of cardiac vagal control is impaired in quiescent UC, but not CD, documented by the increased heart rate, reduced indices of heart rate variability, including pulse interval standard deviation (SD), low-frequency (LF) and HF, the decreased heart rate spectrum, the lower baroreflex sensitivity and baroreflex effectiveness compared with healthy controls (9). By contrast, it was reported that the spectral activity in the VLF and LF bands was lower in children with CD than in healthy controls (10). Besides, HRV may be used as an independent predictor of disease aggravation in pediatric CD (11). It was known that there was a direct linkage between HF power and parasympathetic activity. The study by Wang reported that among the HRV parameters, HF displayed the strongest and most consistent negative correlation with inflammatory activity in CD patients (12). The LF component and LF/HF ratio are associated with a sympathetic–parasympathetic balance or sympathetic modulation (6). LF, the parameter mainly reflecting sympathetic activity, has been confirmed to be negatively correlated with the inflammatory marker C-reactive protein (CRP) (13). Meanwhile, it was confirmed that the parasympathetic activity in adult CD patients was lower than that of healthy controls (12). However, the relationship between parasympathetic activity and CD disease activity remains controversial (8). There is also a lack of research exploring the correlation between HRV parameters and endoscopic activity in CD patients.

This study aimed to (1) analyze the relationship between parasympathetic activity and disease activity (both clinical and endoscopic), (2) investigate the correlations between HRV parameters and systemic inflammatory biomarkers, and (3) evaluate the value of HRV as a non-invasive tool for assessing endoscopic disease activity in CD.

## Materials and methods

It was a prospective cohort study. Eighty-four CD patients were enrolled in the First Affiliated Hospital of Nanjing Medical University from January 2022 to December 2022. All participants were older than 18 years. The patients were recruited from hospitalized CD patients in our single-center IBD. Individuals with pre-existing heart disease were excluded. Individuals with endocrine dysfunction and those with systemic inflammatory disease other than CD were excluded. Individuals with known neurological disorders were excluded. In addition, individuals who had taken medications affecting the cardiovascular system and conduction were excluded. The First Affiliated Hospital of Nanjing Medical University approved the study protocol (2022-SR-367). All participants signed an informed consent form.

### Study procedures

Demographic and clinical data were collected from all study subjects. Heart rate and body mass index (BMI) were measured for each participant.

Fasting venous blood samples were collected from all participants in the morning. Blood samples from all CD patients were tested for routine blood tests, CRP, and erythrocyte sedimentation rate (ESR) to assess the degree of inflammation. The neutrophil-to-lymphocyte ratio (NLR), monocyte-to-lymphocyte ratio (MLR), and platelet-to-lymphocyte ratio (PLR) were calculated from the parameters in a routine blood test. Additionally, stool samples were collected from CD patients to measure fecal calprotectin (FC) for evaluating intestinal inflammation.

Clinical disease activity was evaluated by the Crohn’s Disease Activity Index (CDAI), and the patients were divided into active (CDAI≥150) and inactive groups (CDAI<150). A simple endoscopic score for CD (SES-CD) was used to evaluate endoscopic disease activity, and the patients were divided into an active group (SES-CD>2) and an inactive group (SES-CD ≤ 2) (14, 15). Anxiety and depression levels were assessed by the Hospital Anxiety and Depression Scale (HADS) (16).

### HRV testing procedures

An experienced physician completed the HRV examination of all participants in a stationary environment where possible, using the heart rate variation analyzer (WG-MD-ANSA01 produced by Taiwan We-Gene Technology Company). HRV analysis included time-domain HRV and frequency-domain HRV. The machine collected 5 min RR period continuously, and automatically analyzed the data by collecting 5 min of electrocardiogram (ECG) data, and calculating the time-domain and frequency-domain HRV parameters. During the 5-min test, the subject was required to remain relaxed, maintain a comfortable seated posture, avoid talking and moving, stay calm but not fall asleep, and breathe naturally. Several time-domain and frequency-domain HRV parameters were calculated to investigate the autonomic activity. In the time domain, we calculated one HRV parameter: the standard deviation of all normal–normal (SDNN) intervals. In the frequency domain, we calculated three HRV parameters, including the high-frequency HRV band (HF) (0.15–0.40 Hz), the low-frequency HRV band (LF) (0.04–0.15 Hz), and the very low-frequency HRV band (VLF) (<0.04 Hz) (6). HF reflected parasympathetic activity, and LF reflected the comprehensive activity of sympathetic and parasympathetic efferent nerves. The VLF band is linked to physiological processes such as temperature regulation, vascular vasomotor tone, and the renin-angiotensin-aldosterone system (RAAS) (6). The ratio of low frequency to high frequency (LF/HF) was used to assess the balance between sympathetic and parasympathetic activities, applied to estimate sympathetic activity (17).

### Statistical analysis

SPSS software (version 24.0) was used to analyze the data. Frequency and rate were used to represent count data, and mean ± SD was used to represent measurement data. For a single HRV parameter, the t-test and x^2^ test were used for inter-group comparison of various data types. A *p*-value <0.05 was considered statistically significant, and all were bilateral tests. Pearson’s and Spearman’s correlation coefficients were used, respectively, to evaluate bivariate relationships between variables for normally and non-normally distributed variables. The parameters that were significantly different between the two groups (*p* < 0.05) were selected to establish the assessment model and draw the nomogram. Receiver operating characteristic (ROC) analysis was used to assess the discrimination of the nomogram, presented as the area under the curve (AUC). A calibration curve was used to evaluate the accuracy of the nomogram. The clinical utility of the nomogram was illustrated by the decision curve analysis (DCA). Bootstrap validation was used to validate the nomogram internally.

## Results

### Demographic characteristics, clinical characteristics, inflammatory indicators, HADS scores, and HRV findings in CD patients

We divided the 84 patients into clinically active (*n* = 43) and inactive (*n* = 41) groups according to CDAI. The demographic and clinical characteristics of the two groups are shown in Table 1. Patients with active disease were found to have higher platelet (PLT) (*p* = 0.004), erythrocyte sedimentation rate (ESR) (*p* < 0.001), C-reactive protein (CRP) (*p* = 0.036), and fecal calprotectin (FC) (*p* < 0.001) compared with subjects in clinical remission. There was no statistically significant difference in HAD scores between the two groups. Our results demonstrated that SDNN (*p* = 0.006) and HF (*p* = 0.015) in active patients were lower than those of patients in remission, revealing that parasympathetic activity was impaired among patients with clinically active CD.

**Table 1:** Demographic characteristics and clinical characteristics in CD patients.

Characteristics	Clinical inactive (*n* = 41)	Clinical active (*n* = 43)	*P*	Endoscopic inactive (*n* = 40)	Endoscopic active (*n* = 44)	*P*
Sex (male/female)	33/8	22/21	0.005*	29/11	26/18	0.197
Age [year, M (P25, P75)]	30.0 (25.0, 39.0)	36.0 (24.0, 51.0)	0.215	31.0 (25.5, 44.0)	31.5 (23.0, 49.5)	0.816
BMI [Kg/m2, x ± s]	21.46 ± 3.09	19.90 ± 3.29	0.028*	20.94 ± 3.13	20.41 ± 3.41	0.463
Course of disease [months, M (P25, P75)]	24.0 (10.0, 72.0)	12.0 (3.5, 40.0)	0.043*	24.0 (9.5, 72.0)	12.0 (3.5, 46.0)	0.094
Montreal classification						
Age at diagnosis			0.215			0.852
A1 (≤16) (*n*, %)	6 (14.6%)	3 (7.0%)		4 (10.0%)	5 (11.4%)	
A2 (17 ~ 40) (n, %)	27 (65.9%)	25 (58.1%)		26 (65.0%)	26 (59.1%)	
A3 (>40) (*n*, %)	8 (19.5%)	15 (34.9%)		10 (25.0%)	13 (29.5%)	
Location			0.819			0.524
L1 (ileal) (*n*, %)	15 (36.6%)	13 (30.2%)		16 (40.0%)	12 (27.3%)	
L2 (colonic) (*n*, %)	1 (2.4%)	1 (2.3%)		1 (2.5%)	1 (2.3%)	
L3 (ileocolonic) (*n*, %)	25 (61.0%)	29 (67.4%)		23 (57.5%)	31 (70.5%)	
L4 (upper) (*n*, %)	8 (19.5%)	9 (20.9%)	0.872	8 (20.0%)	9 (20.5%)	0.959
Behavior			0.584			1.000
B1 (non-stricturing, non-penetrating) (*n*, %)	15 (36.6%)	14 (32.6%)		14 (35.0%)	15 (34.1%)	
B2 (stricturing) (*n*, %)	21 (51.2%)	26 (60.5%)		22 (55.0%)	25 (56.8%)	
B3 (penetrating) (*n*, %)	5 (12.2%)	3 (7.0%)		4 (10.0%)	4 (9.1%)	
Perianal disease modifier (*n*, %)	15 (36.6%)	19 (44.2%)	0.478	15 (37.5%)	19 (43.2%)	0.596

^*^p < 0.05.

Meanwhile, we divided the 84 patients into endoscopically active (*n* = 44) and inactive (*n* = 40) groups according to SES-CD. The clinical characteristics of the two groups were similar (Table 1). Higher MLR level (*p* = 0.02), PLR level (*p* = 0.003), PLT level (*p* < 0.001), FC level (*p* < 0.001), ESR level (*p* < 0.001), and CRP level (*p* = 0.02) were found in active group patients, which indicated the presence of an active systemic inflammatory response. Anxiety and depression levels were similar between these two groups. Active CD patients had significantly higher HR (*p* = 0.003) and LF/HF (*p* < 0.001) than patients in remission. In addition, our results demonstrated that SDNN (*p* = 0.002) and HF (*p* = 0.011) in active patients were lower than those in the remission stage, while no statistical difference in LF (*p* = 0.209) and VLF (*p* = 0.054) (Table 2).

**Table 2:** Inflammatory indicators, HADS scores, and HRV findings in CD patients.

Parameters	Clinical inactive (*n* = 41)	Clinical active (*n* = 43)	*P*	Endoscopic inactive (*n* = 40)	Endoscopic active (*n* = 44)	*P*
WBC [10^9/L, x ± s]	5.60 ± 2.44	5.59 ± 1.64	0.979	5.39 ± 2.38	5.79 ± 1.71	0.38
N [10^9/L, x ± s]	3.43 ± 2.20	3.51 ± 1.34	0.835	3.28 ± 2.15	3.65 ± 1.41	0.35
NLR	2.43 ± 1.62	2.83 ± 1.45	0.238	2.42 ± 1.64	2.83 ± 1.42	0.23
MLR	0.32 ± 0.18	0.38 ± 0.18	0.151	0.31 ± 0.15	0.40 ± 0.20	0.02*
PLR	156.36 ± 71.44	224.24 ± 131.16	0.004**	154.38 ± 74.13	224.50 ± 128.14	0.003**
PLT [10^9/L, x ± s]	217.12 ± 64.59	272.33 ± 103.17	0.004**	207.92 ± 65.39	279.43 ± 96.93	<0.001***
ESR [mm/h, x ± s]	14.33 ± 17.57	34.57 ± 30.64	<0.001***	315.04 ± 358.72	707.96 ± 382.61	<0.001***
CRP [mg/L, x ± s]	7.17 ± 16.21	16.49 ± 16.76	0.036*	13.21 ± 20.14	35.12 ± 28.33	<0.001***
FC [μg/g, x ± s]	282.59 ± 302.43	737.54 ± 392.91	<0.001***	6.44 ± 16.41	16.86 ± 16.23	0.02*
HAD-A	3 (0.5)	3 (0.5)	0.656	3 (0, 5)	2.5 (0, 8)	0.591
HAD-D	4 (0.8)	2 (0.5)	0.179	3 (0, 8)	2 (0, 6)	0.306
HR [times/min, x ± s]	76.46 ± 10.28	84.05 ± 11.11	0.002^**^	76.55 ± 9.54	83.80 ± 11.84	0.003**
SDNN [ms, x ± s]	46.99 ± 22.18	35.09 ± 16.12	0.006^**^	47.73 ± 22.0	34.69 ± 15.83	0.002**
LF [ms^2^, x ± s]	631.29 ± 801.40	377.00 ± 474.57	0.079	598.98 ± 805.03	413.98 ± 494.85	0.209
HF [ms^2^, x ± s]	494.02 ± 673.87	214.12 ± 233.51	0.015^*^	506.65 ± 670.02	209.00 ± 252.17	0.011*
VLF [ms^2^, x ± s]	1015.49 ± 1129.92	601.81 ± 766.32	0.052	1018.53 ± 1145.23	608.45 ± 757.15	0.054
LF/HF [%, x ± s]	1.75 ± 1.17	2.35 ± 2.12	0.112	1.38 ± 0.78	2.67 ± 2.12	<0.001***

*p < 0.05; **p < 0.01; ^***^p < 0.001.

### Correlation between diminished parasympathetic activity and disease activity scores, inflammatory biomarkers

At the same time, HRV parameters were significantly correlated with clinical and endoscopic activity scores. SDNN and HF were negatively correlated with the SES-CD score, whereas HR was positively correlated with the SES-CD score. Our results indicated a stronger correlation between endoscopic disease activity and parasympathetic activity (Table 3).

**Table 3:** Correlation between HRV parameters and disease activity scores.

HRV	CDAI		SES-CD	
	*r*	*P*	*r*	*P*
HR	0.406	<0.001^***^	0.388	<0.001^***^
SDNN	−0.270	0.013^*^	−0.325	0.003^**^
HF	−0.122	0.267	−0.395	<0.001^***^
LF	−0.222	0.042^*^	−0.213	0.413
VLF	−0.211	0.054	−0.363	0.001^**^
LF/HF	0.001	0.99	0.292	0.007^**^

*p < 0.05; **p < 0.01; ^***^p < 0.001.

Several correlations were identified between white blood cell count (WBC), neutrophil count (N), neutrophil to lymphocyte ratio (NLR), monocyte to lymphocyte ratio (MLR), platelet to lymphocyte ratio (PLR), FC, ESR, and CRP with various HRV parameters as outlined (Table 4). Of particular importance, a significant inverse correlation was determined between parasympathetic activity (HF and SDNN) and inflammatory activity. These findings suggest a close link between diminished parasympathetic activity and heightened systemic inflammation in CD.

**Table 4:** Correlation between HRV parameters and inflammatory biomarkers.

Parameters	HR	*P*	SDNN	*P*	LF	*P*	HF	*P*	VLF	*P*	LF/HF	*P*
WBC	0.232	0.034^*^	−0.159	NS	−0.063	NS	−0.248	0.023^*^	−0.084	NS	0.188	NS
N	0.156	NS	−0.255	0.019^*^	−0.162	NS	−0.377	<0.001^**^	−0.143	NS	0.278	0.01^*^
NLR	−0.039	NS	−0.304	0.005^**^	−0.245	0.024^*^	−0.379	<0.001^**^	−0.173	NS	0.233	0.033^*^
MLR	−0.081	NS	−0.334	0.002^**^	−0.249	0.022^*^	−0.361	0.001^**^	−0.244	0.025^*^	0.21	NS
PLR	0.008	NS	−0.259	0.017^*^	−0.244	0.025^*^	−0.236	0.03^*^	−0.16	NS	0.087	NS
PLT	0.235	0.031^*^	−0.113	NS	−0.094	NS	−0.123	NS	−0.001	NS	0.092	NS
FC	0.239	NS	−0.157	NS	−0.14	NS	−0.203	NS	−0.262	0.035^*^	0.081	NS
ESR	0.352	0.001^**^	−0.319	0.003^**^	−0.331	0.002^**^	−0.319	0.004^**^	−0.229	0.039^*^	−0.042	NS
CRP	0.091	NS	−0.144	NS	−0.161	NS	−0.224	NS	−0.153	NS	0.183	NS

*p < 0.05; **p < 0.01; ^***^p < 0.001.

### The HRV parametric regression model

**Figure 1:**
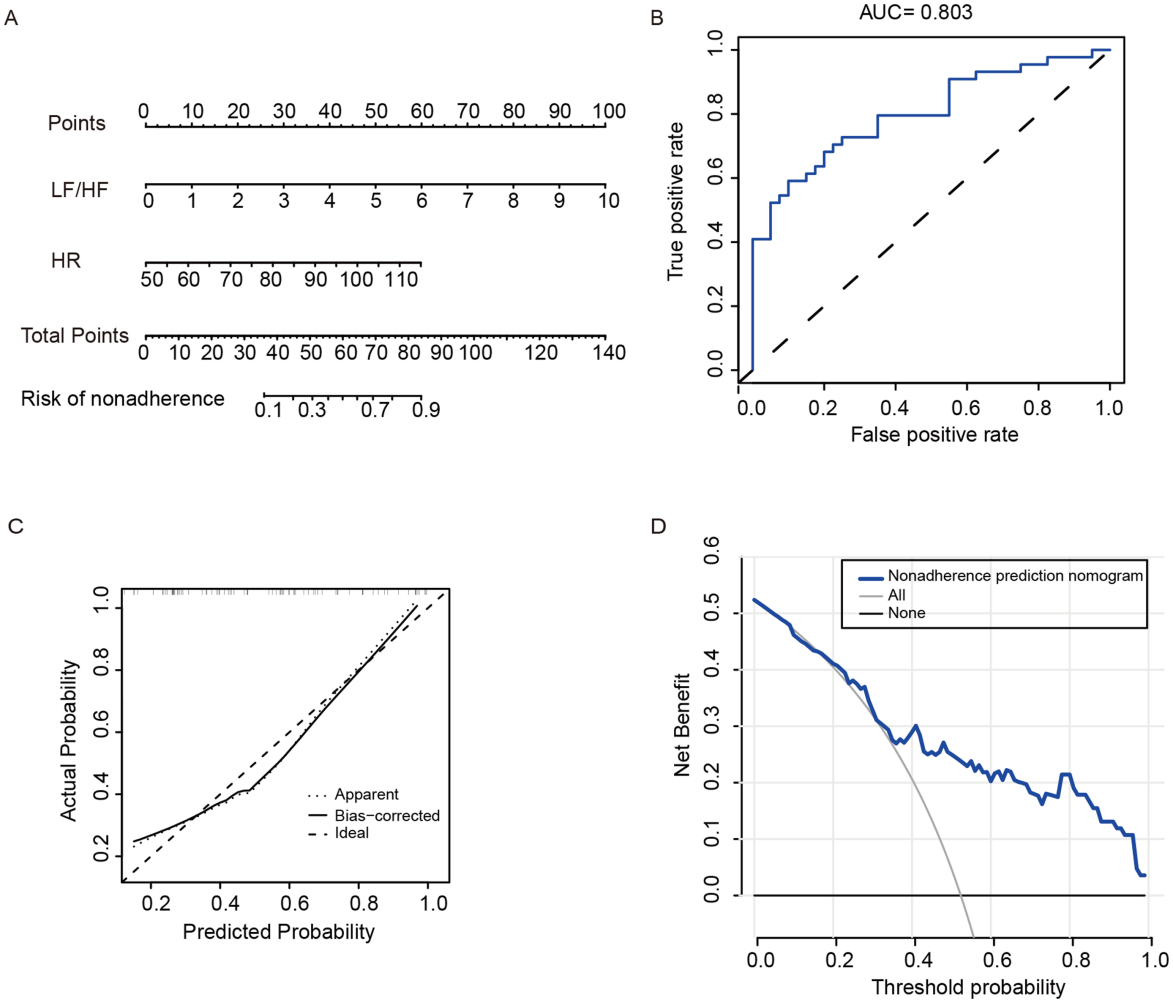
HRV parametric regression model. **(A)** Developed a nomogram based on HRV examination. **(B)** Receiver operating curve of the nomogram. **(C)** Calibration plots of the nomogram. **(D)** Decision curve analyses of the nomogram.

The regression model of endoscopic disease activity was established based on two parameters (HR and LF/HF, R^2^ = 0.375, *p* < 0.001), presented as a nomogram (Figure 1A): HRV assessment of endoscopic disease active = −8.335 + 0.918LF/HF + 0.084HR. The nomogram exhibited sense discrimination, with an AUC of 0.803 (Figure 1B), and the calibration curve proved excellent accuracy (mean absolute error = 0.038) (Figure 1C). The probability was close to the actual probability in the Hosmer–Lemeshow test (*p* = 0.992). It was shown that the nomogram could be used to assess the endoscopic disease activity and provide clinical net benefit if the risk threshold is between 5 and 95% in the DCA (Figure 1D). Meanwhile, bootstrap validation proved the robustness of this model (AUC = 0.791).

## Discussion

HRV is a non-invasive method to evaluate ANS activity, reflecting variations in heartbeat over a specific time period and expressed using time and frequency spectrum methods (6). It analyses variation in the beat-to-beat intervals of the heart and reflects the parasympathetic and sympathetic nervous systems (18). In previous clinical studies, HRV was used to reflect the relationship between chronic gastrointestinal inflammation and autonomic instability (7, 8). Therefore, our study used HRV to reflect autonomic activity in patients with CD and explored the relationship between ANS and inflammation. In the present study, we compared HRV parameters between patients with active and remission CD, and found that patients with active CD showed significant differences in several HRV parameters compared to those in remission. Specifically, SDNN and HF were lower in active patients. Given that HF power has been correlated with parasympathetic activity in previous studies, our findings suggest diminished parasympathetic activity in active CD patients. Furthermore, the increased LF/HF ratio observed in endoscopically active patients may indicate a shift toward relative sympathetic predominance in the autonomic balance. Furthermore, HF is significantly negatively correlated with inflammatory biomarkers and disease activity scores, reflecting reduced parasympathetic nervous system activity in active CD patients. Thereby, we established a model using HRV parameters to assess endoscopic activity in patients with CD.

Earlier studies have reported the impairment of the ANS in patients with CD, while the conclusion remains controversial. We compared HRV findings in patients with active CD and those in remission, and found that SDNN and HF in active patients were lower than those in remission, revealing reduced parasympathetic activity among patients with active CD, which is consistent with a previous study (12). As previously reported, compared with subjects in remission, the parasympathetic activity of patients with CD with active disease was reduced, and it was associated with elevated inflammatory markers (13). Furthermore, it was reported that the parasympathetic activity is associated with anti-inflammatory action, and activation of the vagal efferent stimulates acetylcholine release and attenuates the pro-inflammatory cytokines production, thus alleviating intestinal inflammation (19, 20).

In addition, our study analyzed the correlation between inflammatory biomarkers and HRV parameters. We demonstrated a significant inverse correlation between parasympathetic activity (HF, SDNN) and inflammatory biomarkers (ESR, NLR, MLR, and PLR). This observation strengthened the pathophysiological link between the cholinergic anti-inflammatory pathway and intestinal inflammation in CD. The reduction in parasympathetic activity (21), as quantified by decreased HF power, coincided with elevated levels of inflammatory markers. This supports the concept that impaired parasympathetic activity may permit or exacerbate a systemic inflammatory response in active CD (22–24). This correlation with conventional biomarkers further validates the potential clinical utility of HRV as a complementary tool in the monitoring of CD patients. Meanwhile, HRV parameters were significantly correlated with clinical and endoscopic activity scores. Consequently, HRV measurement transcends mere assessment of autonomic balance; it may serve as a composite, non-invasive indicator reflecting the underlying inflammatory burden.

Finally, we established a nomogram, with strong diagnostic power, based on the two parameters (LF/HF and HR) to assess endoscopic disease activity. In clinical practice, CD disease activity is usually assessed by blood/stool tests and endoscopic examination. HRV examination is a non-invasive and convenient detection method, according to our results, which may be a potential tool to evaluate the disease condition of CD. However, a large number of samples are still needed, along with data on factors such as breathing patterns, drug use, sleep disturbances, and emotional stress, which influence HRV results, to improve the model’s effectiveness.

In addition, stress affects physiological gastrointestinal functions (25). The incidence of anxiety and depression in IBD patients is higher than that in healthy people, which may affect HRV (26). In our study, there was no significant difference in psychological scores between active and remission patients. Nevertheless, we cannot ignore the emotional effect on their HRV in CD patients. Additional psychological scales need to be measured in future studies.

Thus, parasympathetic nerve stimulation must benefit these patients (27–29). Low-intensity stimulation of the parasympathetic nerve reduces visceral pain (30). It was reported that patients with mild to moderate active CD went into remission stage by stimulating the parasympathetic nerve, and the ANS gradually returned to normal or near-normal levels (31).

Our study categorized CD patients based on clinical and endoscopic activity scores, comparing HRV parameters between patients in active and remission phases both clinically and endoscopically, thereby thoroughly evaluating the relationship between HRV parameters and inflammatory activity in CD patients. It is a prospective study, based on a database of multiple clinical and laboratory variables, and it is the first model using HRV to assess endoscopic activity in CD with good diagnostic validity. Short-term HRV is a simple, stable, and non-invasive tool that may assess CD activity. However, it also has several limitations. First of all, this study is a single-center study with a small number of cases and no external verification. Multicenter studies with large samples are needed to verify our regression model. Furthermore, additional research is required to explore the mechanisms of autonomic activity and disease activity in CD.

In conclusion, we observed significant differences in HRV between active and remission CD patients, with decreased parasympathetic activity in those with active disease. HRV indices were significantly correlated with inflammatory markers. Finally, we developed a regression model based on HRV to assess endoscopic disease activity in CD patients.

## Data Availability

The original contributions presented in the study are included in the article/supplementary material, further inquiries can be directed to the corresponding author/s.
